# Cholecystokinin-2 receptor targeting by [^68^Ga]Ga-DOTA-MGS5 PET/CT in a patient with extensive disease small cell lung cancer

**DOI:** 10.1007/s00259-024-06749-z

**Published:** 2024-05-14

**Authors:** Gianpaolo Di Santo, Giulia Santo, Vladan Martinovic, Dominik Wolf, Andreas Pircher, Anna Sviridenko, Judith Löffler-Ragg, Elisabeth von Guggenberg, Irene Virgolini

**Affiliations:** 1 Department of Nuclear Medicine, Medical University of Innsbruck, Innsbruck, Austria; 2 Department of Experimental and Clinical Medicine, ‘‘Magna Graecia’’ University of Catanzaro, Catanzaro, Italy; 3 Department of Pulmonology, Hospital Natters, Innsbruck, Austria; 4 Internal Medicine V, Hematology and Oncology, Medical University of Innsbruck, Innsbruck, Austria


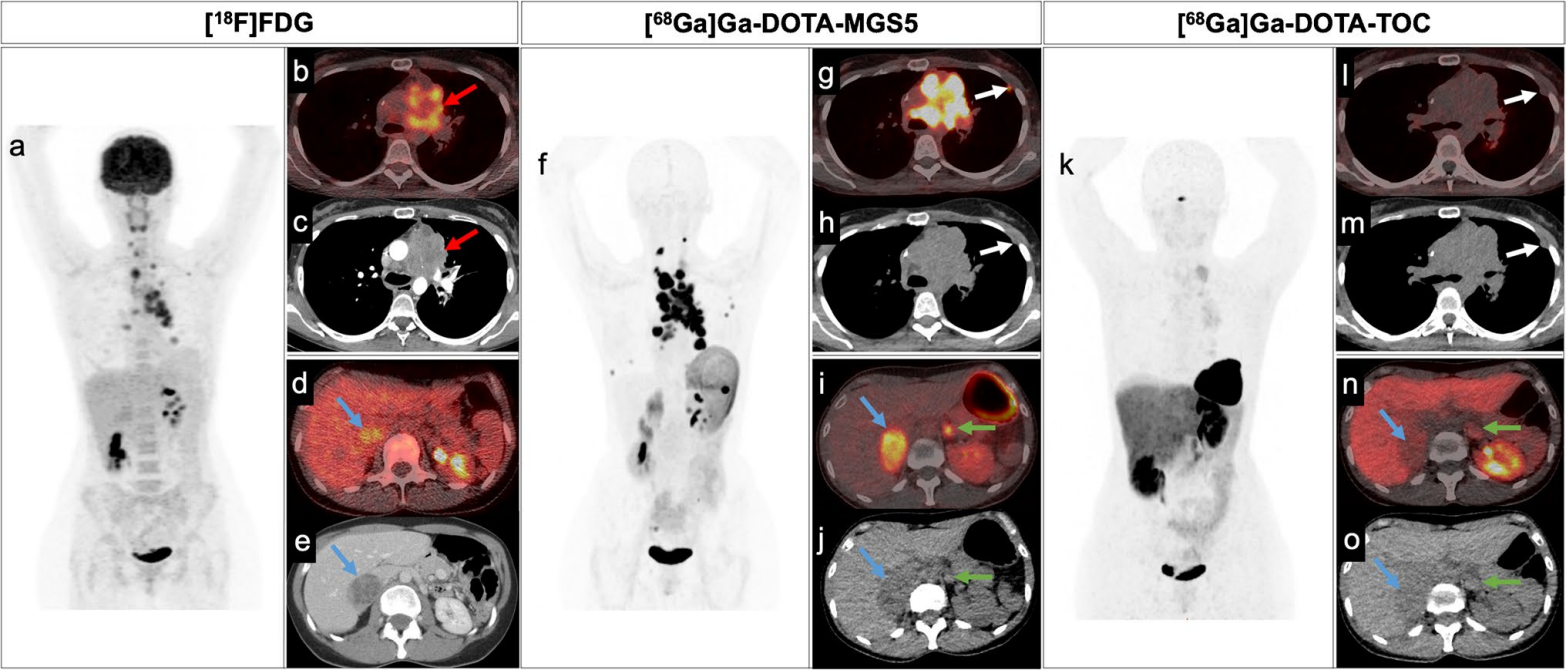


The cholecystokinin-2 receptor (CCK2R) is expressed on various cancer types including small cell lung cancer (SCLC) [1, 2]. Recently, radiolabelled CCK2R-targeting peptides have shown promising results in medullary thyroid carcinoma [3, 4]. We used the minigastrin analogue DOTA-DGlu-Ala-Tyr-Gly-Trp-(N-Me)Nle-Asp-1-Nal-NH_2_ radiolabelled with gallium-68 ([^68^Ga]Ga-DOTA-MGS5) in a 40 year-old patient with extensive disease (ED)-SCLC who also underwent [^18^F]FDG (08/2023) and [^68^Ga]Ga-DOTA-TOC (01/2024) PET/CT. [^68^Ga]Ga-DOTA-MGS5 PET/CT was performed to evaluate the potential therapeutic option with [^177^Lu]Lu-DOTA-MGS5.

[^18^F]FDG PET/CT (a) concentrated in the cervical and mediastinal lymph nodes, the lung tumour and the cervical vertebrae. Inhomogeneous uptake was detected in the mediastinal tumour mass (b) and in the right adrenal gland (d). All lesions were confirmed by contrast-enhanced CT (c,e).

[^68^Ga]Ga-DOTA-MGS5 PET/CT (12/2023), beyond high tracer uptake in all FDG-avid lesions (f), identified additional abnormal foci in left subpleural lesions (g,h), the right mammary region, both adrenal glands (i,j), gastric curvature and the right pelvis. Bone involvement was seen in the left ileum, right VII rib, and cervical vertebrae. These newly detected lesions were not present in the [^18^F]FDG PET/CT suggesting disease progression. Due to the time interval between [^18^F]FDG and [^68^Ga]Ga-DOTA-MGS5 PET/CT scans no direct lesion comparison was performed.

Somatostatin receptor expression as detected by [^68^Ga]Ga-DOTA-TOC PET/CT (k) was faint in all lesions, also in those clearly visible by low-dose CT (l-o).

Considering the poor prognosis of ED SCLC (5-year survival-rate 10–15% [5]), this case suggests that CCK2R-targeting may provide a new theranostic tool in ED-SCLC patients.

## Data Availability

Further data about the case are available from the corresponding authors on reasonable request.
